# *Cordyceps cicadae* Prevents Renal Tubular Epithelial Cell Apoptosis by Regulating the SIRT1/p53 Pathway in Hypertensive Renal Injury

**DOI:** 10.1155/2020/7202519

**Published:** 2020-01-13

**Authors:** Yi Shan Huang, Xu Wang, Zhendong Feng, Hailan Cui, Zebing Zhu, Chenhui Xia, Xueting Han, Wei Jing Liu, Yu Ning Liu

**Affiliations:** ^1^Beijing University of Chinese Medicine, Beijing 100029, China; ^2^Key Laboratory of Chinese Internal Medicine of Ministry of Education, Beijing Dongzhimen Hospital Affiliated to Beijing University of Chinese Medicine, Beijing 100700, China; ^3^Renal Division, Department of Medicine, Peking University First Hospital, Beijing 100034, China; ^4^Renal Division, Beijing Changping Hospital of Traditional Chinese Medicine, Beijing 102200, China; ^5^Department of Endocrinology Nephropathy of Dongzhimen Hospital, Beijing University of Chinese Medicine, Beijing 100700, China

## Abstract

Hypertensive renal injury is a primary etiology of end-stage renal disease, and satisfactory therapeutic strategies are urgently required. *Cordyceps cicadae*, a traditional Chinese herb, has potential renoprotective benefits and is widely used in the treatment of many kidney diseases. To investigate the mechanisms underlying the renoprotective effect of *C. cicadae* on hypertensive renal injury, we studied the effect of *C. cicadae* on tubular epithelial cells (TECs) in a spontaneously hypertensive rat (SHR) model and angiotensin II- (AngII-) cultured primary TECs. Our study showed that *C. cicadae* treatment could decrease 24-hour urine albumin, albumin-to-creatinine ratio (ACR), *β*2-MG level, and kidney injury molecule-1 (kim-1) level in SHR urine, alleviate interstitial fibrosis, and reduce *α*-smooth muscle actin (*α*-SMA) expression in SHR kidney. In primary TECs, medicated serum containing *C. cicadae* (CSM) might significantly reduce the AngII-induced production of kim-1 and neutrophil gelatinase-associated lipocalin (NGAL). Furthermore, *C. cicadae* treatment could decrease TEC apoptosis in SHRs as assessed by the terminal transferase-mediated biotin dUTP nick-end labeling (TUNEL) assay. CSM could inhibit caspase-3 activity and enhance cellular viability as measured by methyl thiazolyl tetrazolium in AngII-cultured TECs, suggesting that CSM might reduce the apoptosis level in TECs induced by AngII. We found that the SIRT1 expression level was markedly lowered, while the protein level of acetylated-p53 was elevated in the TECs of patients with hypertensive renal injury and SHRs. *C. cicadae* presented the effect of regulating the SIRT1/p53 pathway. Further SIRT1 inhibition with EX527 reversed the effect of *C. cicadae* on AngII-induced apoptosis. Taken together, our results indicate that *C. cicadae* offers a protective effect on TECs under hypertensive conditions, which may be related to its antiapoptotic effect through regulation of the SIRT1/p53 pathway.

## 1. Introduction

Hypertensive renal injury is a serious complication of hypertension, which is second to diabetic kidney disease as a key etiology of end-stage renal disease [1]. The pathogenesis of hypertensive renal injury includes activation of the renin-angiotensin-aldosterone system (RAAS), increased arteriosclerosis, elevated sympathetic nervous activity, and water-sodium retention by the kidney [2]. Agents targeting these pathways, such as RAAS blockers and diuretics, may not completely prevent the development of hypertensive kidney injury. In addition, hypotensive drugs may induce the progression of cardiovascular and cardiorenal diseases [3]. Hence, new therapeutic options to improve treatment efficacy are urgently needed.

Tubulointerstitial fibrosis is a typical pathological characteristic of hypertensive renal injury. Recent studies have identified that tubular epithelial cell (TEC) apoptosis plays an important role in the progression of renal tubulointerstitial fibrosis [4–6], as well as in the pathogenesis and progression of hypertensive renal injury [7–9]. However, mechanisms underlying TEC apoptosis in hypertensive renal injury are not fully understood. P53, a tumour suppressor protein, is a key regulator of apoptosis in response to numerous cellular stresses [10]. There is an increasing bulk of evidence supporting the involvement of p53 in TEC apoptosis in many kidney diseases [11–13]. P53 can be activated and stabilized through posttranslational modification pathways, including ubiquitination, phosphorylation, and acetylation [10]. Silent information regulator 2 homolog 1 (SIRT1), a nicotinamide adenine dinucleotide- (NAD^+^-) dependent deacetylase, is widely expressed in TECs and controls multiple diverse processes such as apoptosis, genome stability, stress, and metabolism [14,15]. SIRT1 can inhibit p53 activity through deacetylation, and there is evidence to show that p53 inhibition decreases apoptosis in TECs induced by hyperglycemia, ischemia, and cisplatin [16–19]. However, the role of the SIRT1/p53 pathway in the mechanism of hypertensive renal injury has yet to be examined.

*Cordyceps cicadae (C. cicadae)* is a traditional Chinese herb delivered into the kidney channel and is widely used clinically for the treatment of kidney diseases. *C. cicadae* contains various active ingredients that have potential renoprotective benefits. Cumulative evidence suggests that *C. cicadae* and its active ingredients are effective in ameliorating renal interstitial fibrosis [20–22]. In a previous study, we found that *C. cicadae* could upregulate SIRT1 in TECs and delay the progression of kidney injury in a rat model of diabetic nephropathy [23,24]. Hence, *C. cicadae* could potentially protect TECs from apoptotic injury induced by angiotensin II (AngII) by regulating the SIRT1/p53 pathway. This hypothesis was tested in spontaneously hypertensive rats (SHRs) and AngII-cultured primary TECs in this study.

## 2. Materials and Methods

### 2.1. Patients

All clinical data from 18 patients (aged 30–65 years) at the Affiliated Hospital of Guangdong Medical College were deidentified. Kidney tissue specimens were obtained from patients with biopsy-proven hypertensive renal injury (*n* = 12). The kidney specimens (*n* = 6) obtained from patients with mild urinary protein excretion or only hematuria and characterized with a minimal change in histology were used as controls.

### 2.2. Herbal Formation and Components

*C. cicadae* was obtained from Zhejiang BioAsia Pharmaceutical Co., Ltd. (Pinghu, Zhejiang, China). *C. cicadae* was made into medicated feed for feeding rats, and the daily feed contained *C. cicadae* 4 mg/kg. Medicated serum containing *C. cicadae* (CSM) was prepared for the cell experiment. Sprague-Dawley (SD) rats were treated with intraperitoneal injection of *C. cicadae* extract (1 g/ml, 2 ml/d) or distilled water (2 ml/d) once per day for 1 week. Blood samples were collected via the abdominal aorta 1 h after the last treatment, and CSM and control serum were acquired. Resveratrol and EX527 were purchased from Sigma-Aldrich (St. Louis, MO, USA). Ergosterol peroxide (EP) was purchased from ChemFaces (Wuhan, China).

### 2.3. Animal Experiments

The experimental procedures were performed in accordance with the *National Academies Guiding Principles for the Care and Use of Laboratory Animals, 8th edition*, and approved by the ethics committee of Beijing University of Chinese Medicine. Thirty male SHRs (8 weeks old, weighing 170–210 g) and 8 Wistar–Kyoto (WKY) rats (8 weeks old, weighing 170–210 g) were purchased from Beijing Vital River Co., Ltd. (Beijing, China) and were maintained under controlled lighting, humidity, and temperature conditions with free water access. To exclude the influence of the different daily food intakes of each group on SIRT1 gene expression, the animals were fed similar amounts of food [25]. Blood pressures (BPs) of the SHRs were measured using the tail-cuff method. Rats with BPs maintained at 150–180/90–110 mmHg were included in the next study. Some rats died as the disease progressed. The rest were used in the experiments that followed. Thus, the 4 groups included: (1) the control WKY rats (*n* = 8), (2) SHRs (*n* = 8), (3) SHRs treated with intraperitoneal injection of resveratrol (40 mg/kg/d, *n* = 8), and (4) SHRs treated with *C. cicadae* (4 g/kg/d, *n* = 8). The animals were treated with resveratrol and *C. cicadae* once per day for 20 consecutive weeks. The animals were sacrificed under ether anesthesia. During the experiment, changes in body weight, physical state, and fur condition were observed.

### 2.4. Urinary and Plasmatic Parameters

After overnight fasting in the 28th week, the rats were sacrificed, blood samples were collected via the abdominal aorta, and plasma was prepared. The 24-hour urine was collected from each rat in a metabolic cage at the 28th week. Blood urea nitrogen (BUN), serum creatinine, alanine aminotransferase (ALT), aspartate transaminase (AST), and 24-hour urine protein were measured with enzyme-linked immunosorbent assay (ELISA) kits (C011-2, C013-2, C009-3-1, C010-3-1, and C035-2-1; Nanjing Jiancheng Bioengineering Institute, Nanjing, China). Urinary albumin level was measured using ELISA kits (ab108789; Abcam, Cambridge, MA, USA). Urinary *β*2-MG concentration was measured using ELISA kit (RKM100; R&D Systems, Minneapolis, MN, USA). All operational procedures were performed in accordance with the manufacturers' instruction.

### 2.5. Histological Examination

The paraffin-embedded kidneys were cut into 3 *μ*m sections, dewaxed for 3 × 15 min within the xylene reagent tank, and then rehydrated using ethyl alcohol in a degradation concentration. Hematoxylin-eosin (H&E) staining was performed to observe pathological changes. Masson's trichrome staining (Masson) was applied to each section as reported for fibrosis evaluation. Images were captured using Olympus BX60 microscope (Olympus, Tokyo, Japan) and Zeiss optical microscope with the ZEN 2.3 (blue edition) image capture software. Image-Pro Plus (IPP) 6.0 software (Media Cybernetics, USA) was used to calculate interstitial fibrosis.

### 2.6. Cell Culture and Treatments

Primary human renal proximal tubular epithelial cells (ATCC PCS-400-010, lot 5321) were purchased from ScienCell Research Laboratories, Inc. (Carlsbad, CA., USA) and were maintained in epithelial cell medium (EpiCM; ScienCell Research Laboratories, Inc., Carlsbad, CA., USA). To mimic hypertensive renal injury, the cells were exposed to 10^−7^ mM AngII (Sigma-Aldrich, St. Louis, MO, USA) for 72h. To study the treatment of *C. cicadae*, the cells were cultured in medium containing 10% control serum or 10% CSM. To study the treatment of resveratrol, the cells were exposed to 25 *μ*M resveratrol. Cells were treated with 10 *μ*M EX-527 to inhibit SIRT1 activity. To study the treatment of EP, the cells were exposed to 12.5 *μ*g/ml EP.

### 2.7. Immunohistochemical and Immunofluorescence Studies

Immunostaining analysis of the tissues or cells was performed as described previously [26]. Rabbit anti-SIRT1 for tissue (sc15404; Santa Cruz Biotechnology, Santa Cruz, CA., USA), rabbit antiacetylated-p53 (ab61241; Abcam, Cambridge, MA, USA), rabbit anti-*α*-smooth muscle actin (*α*-SMA) antibody (ab32575; Abcam, Cambridge, MA, USA), and fluorescein isothiocyanate-labeled goat anti-rabbit IgG (sc-2012; Santa Cruz Biotechnology, Santa Cruz, CA, USA) were used in the immunostaining assay for the tissues. Images were taken using the Olympus BX60 microscope (Olympus, Tokyo, Japan) or TCS SP5 II confocal microscope (Leica Microsystems, Wetzlar, Germany), under the same condition. Expression levels of SIRT1 and acetylated-p53 were analyzed with the average optic density using the IPP 6.0 software. Rabbit antiacetylated-p53 (ab61242; Abcam, Cambridge, MA, USA), rabbit anti-SIRT1 for cell (2977886; Millipore, Billerica, MA., USA), and Alexa fluor 488 donkey anti-rabbit IgG (Invitrogen, Carlsbad, CA, USA) were used in the immunostaining for cells. Microscopic images were taken using a TCS SP5 II confocal microscope. The average fluorescence intensity of SIRT1 and acetylated-p53 was analyzed using the IPP 6.0 software. Apoptosis was assessed using a TUNEL apoptosis assay kit (G7130; Promega, Madison, WI, USA) in accordance with the manufacturer's instruction.

### 2.8. Western Blot Analysis

Western blot analysis was performed as described previously [26]. The primary antibodies against SIRT1 for tissue (sc15404; Santa Cruz Biotechnology, Santa Cruz, CA, USA), SIRT1 for cell (2977886; Millipore, Billerica, MA., USA), and acetylated-p53 (ab61242; Abcam, Cambridge, MA, USA), GADPH (10494-1-AP, Proteintech), Tubulin (ab59680; Abcam, Cambridge, MA, USA), and horseradish peroxidase-conjugated secondary antibodies (SA0000I-I; Proteintech, Rosemont, PA., USA) were used.

### 2.9. Capillary Electrophoresis Immunoquantification

For quantitative capillary isoelectric immunoassay, simple western immunoblotting was performed on a WES capillary electrophoresis instrument (ProteinSimple, San Jose, CA, USA) using the size separation master kit with split buffer (12–230 kDa) in accordance with the manufacturer's standard instruction. On the WES system, 3 µl of protein was run using the following antibodies: anti-SIRT1 antibody (sc15404; Santa Cruz Biotechnology, Santa Cruz, CA., USA) and anti-GAPDH antibody (10494-1-AP; Proteintech, Rosemont, PA., USA). All other reagents (antibody diluent, secondary antibodies) were from ProteinSimple. The Compass software (ProteinSimple version 3.1.8) was used to program the WES instrument and to quantify the western immunoblots. Output data were displayed from the software-calculated average of nine exposures (1-512s). The run conditions were as recommended by the manufacturer. Peak areas were determined using the Compass software and normalized to anti-GAPDH.

### 2.10. Cell Viability Assay

Cells were incubated with a 5 mg/ml methyl thiazolyl tetrazolium (MTT) solution (475989; Calbiochem, San Diego, CA., USA) for 4 h at 37°C. The formazan crystals were dissolved in dimethylsulfoxide. Optical density was determined at 570 nm with a plate reader (Multiskan MK3, Thermo LabSystems, Helsinki, Finland).

### 2.11. Caspase-3 Activity Assay of Cells

Cells were treated for 72 h and then lysed in PathScan Sandwich ELISA lysis buffer (1×; 7018, Cell Signaling Technology). Cell lysate was added to the assay plates containing the substrate solution, and plates were incubated at 37°C in the dark. Relative fluorescent units were acquired at 1 h by using a caspase-3 activity assay kit (5723; Cell Signaling Technology, Danvers, MA, USA).

### 2.12. Biochemical and Enzymatic Assays

Levels of neutrophil gelatinase-associated lipocalin (NGAL) and kidney injury molecule (KIM)-1 in rat urine and cell culture supernatants were measured with Quantikine ELISA kits purchased from R&D Systems (DLCN20; Minneapolis, MN, USA) and Abcam (ab119597; Cambridge, MA, USA).

### 2.13. Statistical Analyses

All data were expressed as mean ± standard error of the mean (SEM). A multiple group comparison was performed using one-way analysis of variance and Dunnett's post hoc tests. Data were considered as statistically significant if the *p* value was <0.05. Statistical tests were performed with SPSS 20.0.

## 3. Results

### 3.1. *C. cicadae* Improved the Renal Functions of SHRs

**Figure 1:**
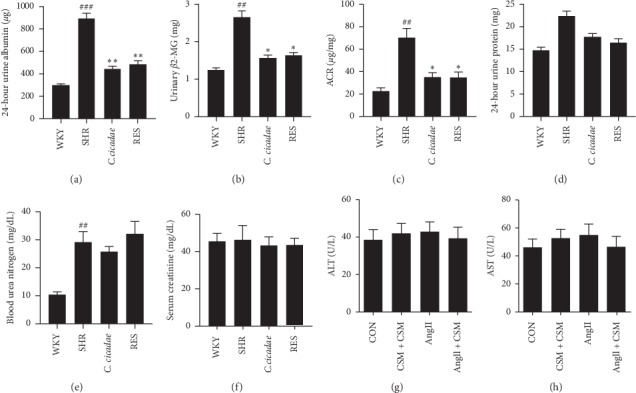
Effects of *Cordyceps cicadae* (*C. cicadae*) and resveratrol (RES) on urinary and plasmatic biochemical parameters in spontaneously hypertensive rats (SHRs). *C. cicadae* treatment significantly reduced 24-hour albuminuria, albumin-to-creatinine ratio (ACR), and *β*2-MG level at the 28th week. *C. cicadae*: SHRs treated with 4 g/kg/d *C. cicadae*; RES : SHRs treated with 40 mg/kg/d resveratrol. ^##^*p* < 0.01 and ^###^*p* < 0.001, as compared with WKY. ^*∗*^*p* < 0.05 and ^*∗∗*^*p* < 0.01, as compared with SHR.

As hypertensive renal injury models, SHRs were used and observed for the effects of *C. cicadae* on body weight, BP, and biochemical markers at the 28th week. Food intake was standardized and limited to 15 g/d to exclude the influence of different daily intakes on SIRT1 gene expression. No significant difference in body weight was found between the groups. BP was remarkably higher in the SHRs than in the WKY rats, while *C. cicadae* treatment had no effect on BP (Table 1). The SHRs exhibited high BUN levels, as well as increased 24-hour urine albumin, albumin-to-creatinine ratio (ACR), and *β*2-MG levels at week 28 (Figures 1(a)–1(c) and 1(e)), while the serum creatinine level and 24-hour urine protein showed no significant difference (Figures 1(d) and 1(f)). *C. cicadae* treatment significantly reduced 24-hour albuminuria, ACR, and *β*2-MG level at the 28th week (Figures 1(a)–1(c)). Liver function markers such as ALT and AST were measured, and no significant difference was observed amongst the groups (Figures 1(g) and 1(h)).

### 3.2. *C. cicadae* Attenuated the Renal Injury Induced by AngII

**Figure 2:**
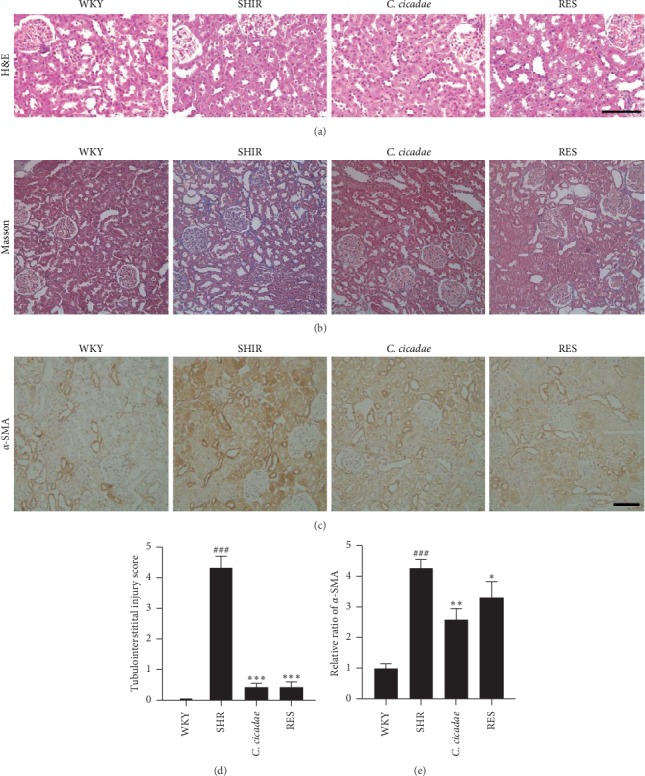
Effects of *Cordyceps cicadae* (*C. cicadae*) and resveratrol (RES) on tubulointerstitial fibrosis and fibrosis-relative protein induced by hypertension. (a) Hematoxylin-eosin (H&E) staining: no distinct pathological differences amongst the groups were observed. (b, d) Masson staining: SHR group showed severe collagen deposition in renal interstitium. *C. cicadae* and RES significantly reduced the deposition of collagen. (c) Immunohistochemical staining of *α*-smooth muscle actin (*α*-SMA) in each group. (e) Average optical intensity of *α*-SMA was measured. The expression of *α*-SMA significantly increased in spontaneously hypertensive rats (SHRs), which was reduced after treatment with *C. cicadae* and RES. Scale bar: 100 *μ*m. *C. cicadae*: SHRs treated with 4 g/kg/d *C. cicadae*; RES : SHRs treated with 40 mg/kg/d resveratrol. ^###^*p* < 0.001, as compared with the WKY, ^*∗*^*p* < 0.05, ^*∗∗*^*p* < 0.01, and ^*∗∗∗*^*p* < 0.001, as compared with SHR.

**Figure 3:**
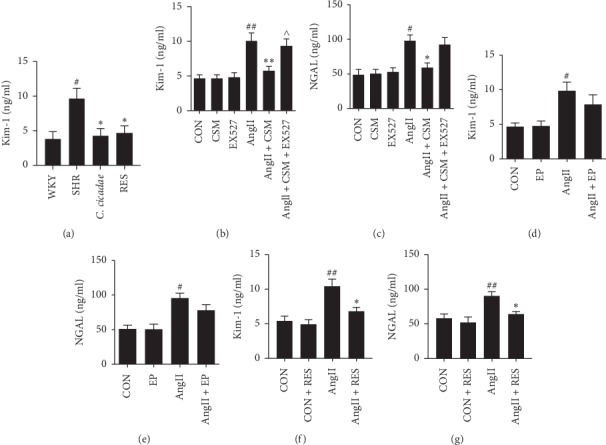
Effects of *Cordyceps cicadae* (*C. cicadae*) and resveratrol (RES) on tubular epithelial cell (TEC) injury induced by angiotensin II (AngII). (a) The level of urinary kim-1 was measured with enzyme-linked immunosorbent assay (ELISA) in the rats in all the groups. *C. cicadae*: spontaneously hypertensive rats (SHRs) treated with 4 g/kg/d *C. cicadae*; RES: SHRs treated with 40 mg/kg/d resveratrol. (b, c) Levels of kim-1 and NGAL were measured with ELISA in culture supernatants of TECs treated with 10% CSM and/or 10 *μ*M EX527 in the presence or absence of 10^−7^ mM AngII. (d–g) Levels of kim-1 and NGAL were measured with ELISA in culture supernatants of TECs. The CSM and RES treatments reduced the secretion of kim-1 and NGAL in the TECs after exposure to AngII, and EX527 reversed the beneficial effect of CSM. EP: TECs treated with 12.5 *μ*g/ml ergosterol peroxide. RES: TECs treated with 25 *μ*M resveratrol. ^#^*p* < 0.05 and ^##^*p* < 0.01, as compared with the WKY or control TECs. ^*∗*^*p* < 0.05 and ^*∗∗*^*p* < 0.01, as compared with SHRs or TECs exposed to AngII. ^∧^*p* < 0.05, as compared with TECs exposed to AngII plus CSM.

H&E and Masson were performed to evaluate the effects of *C. cicadae* on the pathological injury of kidney tissues. No distinct pathological differences among the groups were observed through H&E staining (Figure 2(a)), while Masson staining showed that *C. cicadae* significantly reduced the deposition of collagen in the renal interstitium of SHRs (Figures 2(b) and 2(d)). In addition, the expression of fibrosis-related protein *α*-SMA was significantly increased in SHRs, which was reduced after treatment with *C. cicadae* (Figures 2(c) and 2(e)). Furthermore, renal tubular injury marker kim-1 and NGAL were measured in vivo and in vitro. Urinary kim-1 level increased in the SHRs, which was significantly decreased after *C. cicadae* treatment (Figure 3(a)). In vitro, we treated TECs with CSM and observed the protective effects of CSM on TECs. Firstly, cells were exposed to 5%, 10%, 15%, and 30% CSM or control serum, and MTT assay was performed to assess the cell viability. 5% and 10% CSM presented no distinct effect on the cell viability. Though the concentration of CSM rose to 15% and 30%, it still showed no significant bad effect on cell viability (data not shown). Subsequently, after 72 h of exposure to AngII, the levels of kim-1 and NGAL in primary TEC culture supernatants were significantly increased as compared with those in the control group and were effectively reduced after CSM treatment (Figures 3(b) and 3(c)). Conversely, the major sterol of *C. cicadae*, EP, presented no obvious effect on the reduction of the levels of kim-1 and NGAL (Figures 3(d) and 3(e)).

### 3.3. *C. cicadae* Inhibited the TEC Apoptosis Induced by AngII

**Figure 4:**
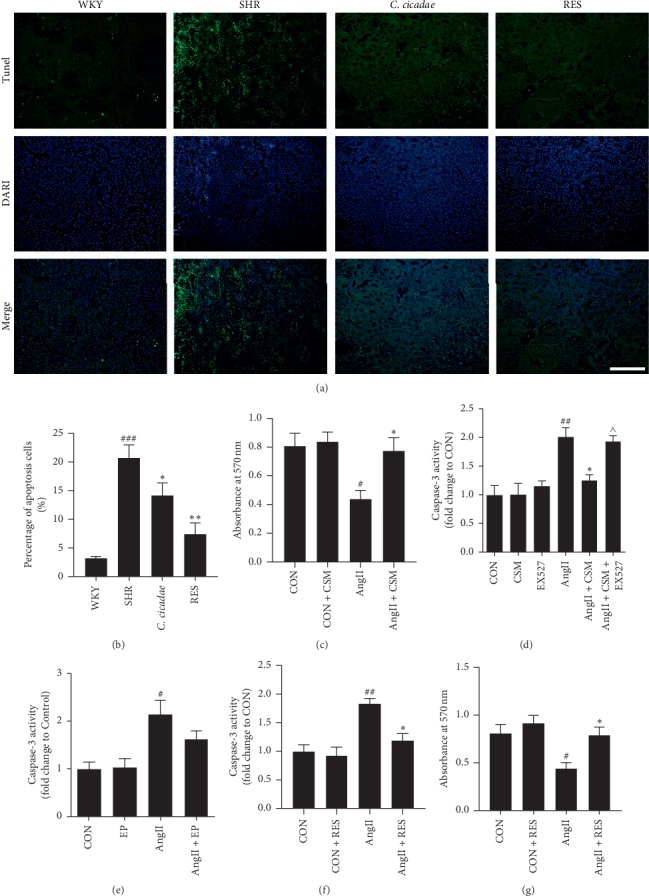
Effects of *Cordyceps cicadae* (*C. cicadae*) and resveratrol (RES) on tubular epithelial cell (TEC) apoptosis in spontaneously hypertensive rats (SHRs) and angiotensin II (AngII)-treated TECs. (a, b) Apoptosis was assessed with terminal transferase-mediated biotin dUTP nick-end labeling (TUNEL) in the renal tubules of the rats in each group. Scale bar: 100 *μ*m. *C. cicadae*: SHRs treated with 4 g/kg/d *C. cicadae*; RES: SHRs treated with 40 mg/kg/d resveratrol. (c, g) Cell viability was assessed with methyl thiazolyl tetrazolium (MTT) assay. (d) Apoptosis was assessed with caspase-3 activity level in culture supernatants of TECs treated with 10% medicated serum containing *C. cicadae* (CSM) and/or 10 *μ*M EX527 in the presence or absence of 10^−7^ mM AngII. (e, f) The caspase-3 activity level was measured amongst the groups. *C. cicadae* and RES significantly inhibited the TEC apoptosis induced by AngII, and EX527 reversed the effect of CSM on the AngII-induced TEC apoptosis. CSM: TECs treated with 10% CSM; EP: TECs treated with 12.5 *μ*g/ml ergosterol peroxide; RES: TECs treated with 25 *μ*M resveratrol. ^##^*p* < 0.01 and ^###^*p* < 0.001, as compared with the WKY or control TECs. ^*∗*^*p* < 0.05 and ^*∗∗*^*p* < 0.01, as compared with the SHR or TECs exposed to AngII. ^∧^*p* < 0.05, as compared with TECs exposed to AngII plus CSM.

To evaluate the apoptosis in TECs induced by AngII, we assessed the number of apoptotic cells in the kidney by TUNEL detection. The number of apoptotic TECs was higher in the SHR group than in the WKY group, which could be significantly reduced by treatment with *C. cicadae* (Figures 4(a) and 4(b)). In addition, exposure to AngII significantly increased TEC apoptosis and inhibited cell viability, which were determined based on caspase-3 protein activity and MTT assay results, respectively. CSM reversed caspase-3 activity and enhanced the cellular viability of primary TECs after exposure to AngII (Figures 4(c) and 4(d)). We also examined the effect of EP on caspase-3 activity, and results showed that there was no significant effect (Figure 4(e)).

### 3.4. Disruption of the SIRT1/p53 Pathway in the TECs of Patients with Hypertensive Renal Injury

**Figure 5:**
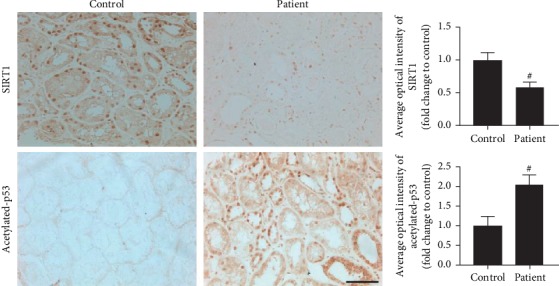
Changes in SIRT1 and acetylated-p53 expression levels in renal tubules from controls and patients with hypertensive renal injury. The expression levels of SIRT1 and acetylated p53 were measured with immunohistochemical assay. Scale bar: 40 *μ*m. Protein levels of SIRT1 were decreased, while the acetylated-p53 levels increased in the renal tubules of patients with hypertensive renal injury as compared with the controls. ^#^*p* < 0.05 as compared with control.

As the SIRT1/p53 pathway was reported to be associated with multiple kidney injuries, we explored the change of the SIRT1/p53 pathway in TECs of hypertensive renal injury patients and SHRs. Kidney specimens were collected from control subjects and patients with hypertensive renal injury. We used immunohistochemical technology to examine the expressions of SIRT1 and acetylated-p53. As shown in Figure 5, lower SIRT1 expression and more acetylated-p53-positive puncta were found in renal TECs from patients with hypertensive renal injury than in the control group.

### 3.5. *C. cicadae* Normalized the SIRT1/p53 Pathway in the TECs of SHRs and AngII-Cultured Primary TECs

**Figure 6:**
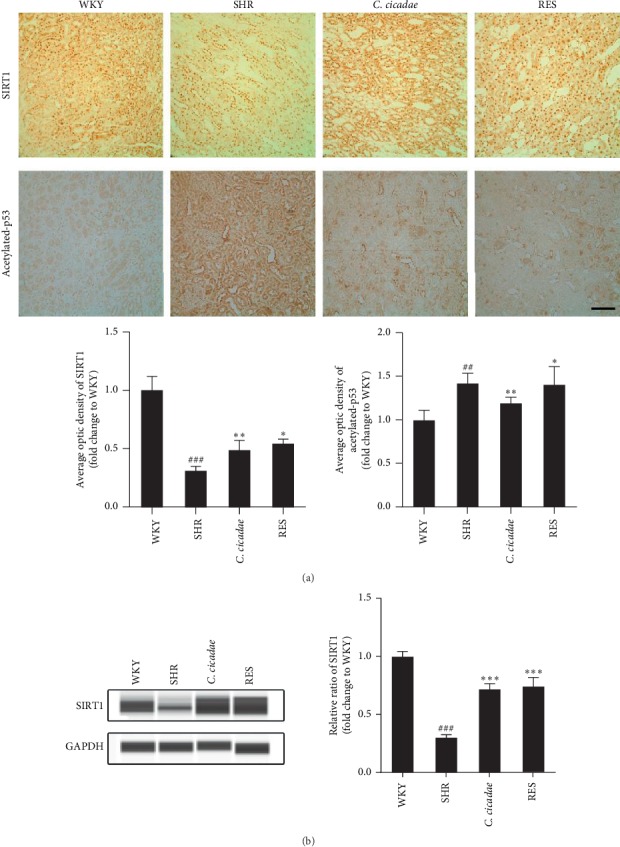
Effects of *Cordyceps cicadae* (*C. cicadae*) and resveratrol (RES) on the expression levels of SIRT1 and acetylated-p53 in tubular epithelial cells (TECs) of spontaneously hypertensive rats (SHRs). (a) Immunohistochemical staining for SIRT1 and acetylated p53 in the renal tubules of the rats in each group. Scale bar: 100 *μ*m. The optical intensity of the SIRT1 and acetylated p53 was measured. (b) Capillary electrophoresis immunoquantification of SIRT1 in the renal cortex of the rats in each group. Densitometry was performed for quantification, and the ratio of SIRT1 to GAPDH was expressed as a fold of the control. *C. cicadae*: SHRs treated with 4 g/kg/d *C. cicadae*; RES : SHRs treated with 40 mg/kg/d resveratrol. *C. cicadae* increased SIRT1 expression and decreased p53 acetylation in the renal tubules of the SHRs, similar to the effects of resveratrol. ^##^*p* < 0.01 and ^###^*p* < 0.001, as compared with the WKY. ^*∗*^*p* < 0.05, ^*∗∗*^*p* < 0.01, and ^*∗∗∗*^*p* < 0.001, as compared with the SHR.

**Figure 7:**
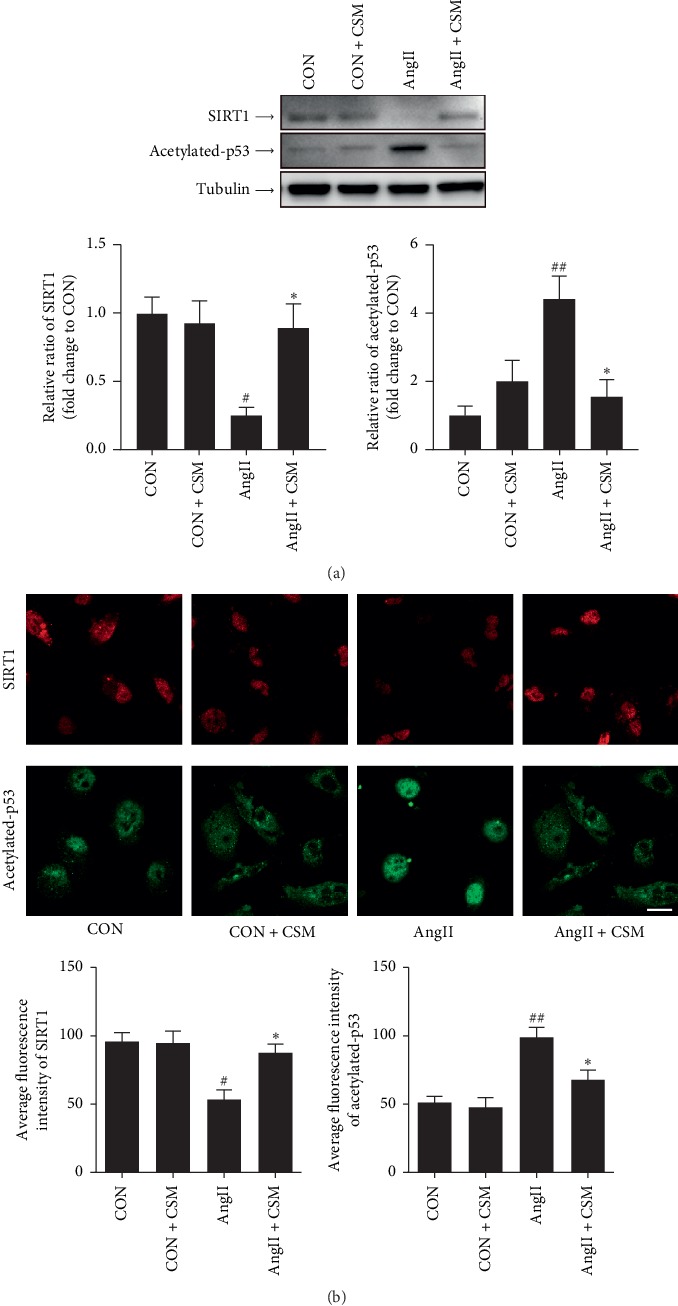
Effects of medicated serum containing *Cordyceps cicadae* (CSM) on SIRT1 and acetylated p53 in tubular epithelial cells (TECs) treated with angiotensin II (AngII). (a) Western blot analysis of SIRT1 and acetylated p53 in the TECs exposed to 10^−7^ mM AngII or vehicle treated with CSM or control serum. Densitometry was performed for quantification, and ratios of SIRT1 and acetylated-p53 to tubulin were expressed as a fold of the control. (b) Immunofluorescent staining for SIRT1 (red) and acetylated p53 (green) in TECs as described in (a). Scale bar: 20 *μ*m. Average fluorescence intensity was performed for quantification. CSM upregulated SIRT1 expression in the AngII-treated TECs and downregulated p53 acetylation. ^#^*p* < 0.05 and ^##^*p* < 0.01, as compared with the control TECs. ^*∗*^*p* < 0.05 as compared with the TECs exposed to AngII.

We found that *C. cicadae* effectively attenuated TEC injury induced by AngII. We then studied if the renoprotective function of *C. cicadae* was related to SIRT1/p53 pathway regulation. As shown in Figure 6, *C. cicadae* treatment significantly enhanced SIRT1 expression and suppressed acetylated-p53 expression, which suggests that *C. cicadae* could inhibit p53 acetylation through a SIRT1-dependent pathway. In addition, we confirmed these results in AngII-cultured TECs and obtained consistent results (Figure 7).

### 3.6. SIRT1 Inhibition Reversed the Protective Effect of Resveratrol on AngII-Induced Apoptosis in TECs

To confirm that the effect of *C. cicadae* is associated with a SIRT1-dependent mechanism, we conducted an experiment using a SIRT1 inhibitor, EX527. EX527 significantly reversed the effect of CSM on the AngII-induced TEC apoptosis, which was assessed based on caspase-3 protein activity (Figure 4(d)). In addition, SIRT1 inhibition by EX527 significantly blocked the effect of CSM on the reduction of kim-1 level. Also, the effect of CSM on the reduction of NGAL tended to be reversed by EX527, although there was no significant difference (Figures 3(b) and 3(c)).

### 3.7. SIRT1 Activator Protected TECs from AngII-Induced Apoptosis

P53 is a tumour suppressor protein that initiates multiple apoptotic pathways, while SIRT1 can inhibit its activity through deacetylation. To further examine the effects of SIRT1/p53 pathway regulation on TEC injury induced by AngII, we use resveratrol, a potent SIRT1-activating molecule, to upregulate the expression of SIRT1. Resveratrol treatment successfully increased SIRT1 expression and decreased acetylated-p53 expression in the TECs of SHRs (Figure 6). Resveratrol treatment significantly reduced TEC apoptosis in the SHRs (Figures 4(a) and 4(b)), as assessed using the TUNEL assay. Consistent results were obtained in vitro. Resveratrol inhibited cellular apoptosis and enhanced cell viability, which were deduced based on caspase-3 protein activity and MTT assay results, respectively (Figures 4(f) and 4(g)). In terms of renal injury, resveratrol reduced the deposition of collagen in renal interstitium and the expression of *α*-SMA of SHRs (Figure 2). In addition, resveratrol reduced the increased urinary kim-1 level of the SHRs, as well as the enhanced kim-1 and NGAL secretions induced by AngII in the primary TECs (Figures 3(f) and 3(g)).

## 4. Discussion

In this study, we demonstrated that *C. cicadae* effectively reduced 24-hour urine albumin, ACR, and *β*2-MG excretion in the SHRs and also the level of TEC apoptosis induced by AngII in vivo and in vitro. This therapeutic effect of *C. cicadae* is associated with the regulation of the SIRT1/p53 pathway.

Renal tubular dysfunction is the first manifestation of the progression of hypertensive renal injury. Various lesions in kidney tubules, ranging from TEC flattening and dilatation to tubular atrophy and loss, may result in hypertensive kidney injury and subsequently tubulointerstitial fibrosis [27,28]. In this study, our findings are consistent with these viewpoints. The expression levels of the tubular injury markers, such as urinary *β*2-MG, kim-1, and NGAL, were significantly increased in the SHRs as compared with the WKY rats. There was no significant difference in serum creatinine levels between WKY and SHR groups, indicating that glomerular function of the experimental animals was not seriously damaged. Previous studies showed that proximal renal tubular cells largely take up filtered albumin [29,30], and decreased reabsorption of protein in proximal tubules promotes proteinuria in SHRs [31]. Hence, increased 24-hour urine albumin and ACR may be induced by proximal tubule injury.

TEC apoptosis is an important cause of tubular atrophy and renal tubulointerstitial fibrosis [5]. AngII could induce TEC apoptosis both in vivo and in vitro via the activation of RAAS, oxidative stress, and inflammation [7–9]. However, the underlying mechanisms require further elucidation. In our study, *C. cicadae* inhibited AngII-induced TEC apoptosis and alleviated interstitial fibrosis. As *C. cicadae* had no effect on the BPs of the SHRs, this suggests that AngII might contribute to TEC apoptosis in SHRs through a BP-independent pathway.

P53 is a tumour suppressor gene that initiates multiple apoptotic pathways via both transcriptional dependent and independent functions. Posttranslational modifications such as ubiquitination, phosphorylation, and acetylation can regulate the subcellular localization, stability, and activity of p53 [10]. P53 is a crucial nonhistone substrate of the NAD^+^-dependent deacetylase SIRT1 [15]. SIRT1 prevents p53 nuclear translocation and blocks p53 transcription-dependent apoptosis by inhibiting p53 acetylation. A previous study reported that AngII degrades SIRT1, hence promoting p53 acetylation and inducing apoptosis in cardiomyocytes [32]. SIRT1 is an important regulator that provides renoprotective effects against various renal disorders, but its role in hypertensive renal injury has rarely been studied. Thus, we established a hypertensive renal injury model to explore the role of the SIRT1/p53 pathway in the apoptosis of TECs. Our results showed that AngII degraded SIRT1, which increased p53 acetylation, and subsequently enhanced apoptosis in the TECs.

Resveratrol has been recognized as a potent SIRT1-activating molecule since 2003 [33]. Though recent reports indicate that resveratrol may not be a SIRT1-specific activator, the beneficial effects of resveratrol are thought to be mediated in large by the activation of SIRT1 [34]. In addition, the SIRT1-activating effect of resveratrol is dose-dependent. Recent studies reported that lower doses of resveratrol (≤25 *μ*M in vitro, ≤50 mg/kg in vivo, generally) resulted in SIRT1-dependent effects, while higher doses led to other functions independent of SIRT1 [35,36]. In our study, we used resveratrol as a SIRT1 activator at a lower dose of 40 mg/kg in vivo and 25 *μ*M in vitro. After treatment with resveratrol, we observed overexpressed SIRT1, as well as reduced p53 acetylation and apoptotic level in the TECs. We found that *C. cicadae* presented a similar effect as resveratrol, and the inhibitor of SIRT1 and EX527, reversed the renal beneficial effects of *C. cicadae*, which indicates that the renoprotective effects of *C. cicadae* might be via an SIRT1-dependent mechanism.

*C. cicadae* is a traditional Chinese herb that has been used in China for more than 1,000 years. It contains various biologically active chemical substances such as ergosterol peroxide (EP), N6-(2-hydroxyethyl) adenosine, polysaccharides, cordycepic acid, and effective nucleosides [37], which play a comprehensive role in renal protection through their anti-inflammatory and antifibrotic effects. EP is the major sterol produced by *C. cicadae*. In our study, EP presented no significant effects on AngII-induced injury in TECs, which indicates that the renal benefits of *C. cicadae* might depend on the combined effect of several compounds. *C. cicadae* is widely used in the clinical treatment of chronic kidney disease and has low toxicity and few side effects, indicating its safe usage. These findings may offer a new therapeutic strategy for treating hypertensive renal injury.

## 5. Conclusion

In summary, this study proved that *C. cicadae* has renoprotective effects in a rat model with hypertensive renal injury and primary TEC model after exposure to AngII. *C. cicadae* can also regulate the SIRT1/p53 pathway and has an antiapoptotic effect in TECs. The inhibition of SIRT1 reversed the renal beneficial effects of *C. cicadae*, Therefore, we conclude that the renoprotective function of *C. cicadae* is probably attributable to its antiapoptotic effect by regulating the SIRT1/p53 pathway, which may offer a new therapeutic strategy for treating hypertensive renal injury.

## Figures and Tables

**Table 1:** Effects of *Cordyceps cicadae* (*C. cicadae*) and resveratrol (RES) on body weight, systolic blood pressure (BP), and diastolic BP in Wistar–Kyoto (WKY) rats and spontaneously hypertensive rats (SHRs).

	WKY	SHR	*C. cicadae*	RES
Body weight (g)	365 ± 17	348 ± 30	343 ± 11	341 ± 30
Food intake (g/d)	15	15	15	15
Systolic BP (mmHg)	123 ± 17	178 ± 9^###^	171 ± 10	179 ± 14
Diastolic BP (mmHg)	101 ± 14	127 ± 19^##^	140 ± 9	143 ± 13

Data are expressed as mean ± SEM. ^##^*p* < 0.01 and ^###^*p* < 0.001, as compared with WKY. *C. cicadae*: SHRs treated with 4 g/kg/d *C. cicadae*; RES : SHRs treated with 40 mg/kg/d resveratrol.

## Data Availability

The data used to support the findings of this study are available from the corresponding author upon request.
